# Changing the Treatment Paradigm for Hepatocellular Carcinoma Using Atezolizumab plus Bevacizumab Combination Therapy

**DOI:** 10.3390/cancers13215475

**Published:** 2021-10-30

**Authors:** Masatoshi Kudo

**Affiliations:** Department of Gastroenterology and Hepatology, Faculty of Medicine, Kindai University, Osaka 589-8511, Japan; m-kudo@med.kindai.ac.jp; Tel.: +81-72-366-0221 (ext. 3149)

**Keywords:** hepatocellular carcinoma, atezolizumab plus bevacizumab, ABC conversion therapy

## Abstract

**Simple Summary:**

A phase 3 IMbrave150 trial showed that atezolizumab plus bevacizumab combination therapy had an improved survival benefit over sorafenib. An excellent direct anti-cancer effect, including progression-free survival and objective response rate, was also observed with this combination therapy. It also showed very favorable effects in patients who had poor prognoses, with main portal vein invasion, tumor occupancy ≥50%, or biliary tract invasion. The liver function was determined through the albumin–bilirubin score, which was well-maintained throughout the treatment period. Patients reported excellent outcomes in terms of quality of life. With these favorable features, the treatment paradigm for hepatocellular carcinoma was drastically changed by atezolizumab plus bevacizumab combination therapy. This was especially observed in intermediate-stage hepatocellular carcinoma, where cancer-free and drug-free status was achieved through the switch to a curative therapy such as resection, ablation, or curative transarterial chemoembolization. This review covers these important issues in this paradigm shift, in addition to recently raised and debated issues, such as its response to tumors with WNT/β-catenin mutations and non-alcoholic steatohepatitis-related hepatocellular carcinoma.

**Abstract:**

Atezolizumab plus bevacizumab combination therapy was approved worldwide for use in 2020. A 30% objective response rate with 8% complete response (CR) was achieved in a phase 3 IMbrave150 trial. Here, the change in the treatment strategy for hepatocellular carcinoma (HCC) using atezolizumab plus bevacizumab combination therapy is reviewed. The phase 3 IMbrave150 clinical trial was successful because of the direct antitumor effect of bevacizumab, which shifted the suppressive immune microenvironment to a responsive immune microenvironment, in addition to its synergistic effects when combined with atezolizumab. The analysis of CR cases was effective in patients with poor conditions, particularly tumor invasion in the main portal trunk (Vp4), making the combination therapy a breakthrough for HCC treatment. The response rate of the combination therapy was 44% against intermediate-stage HCC. Such a strong tumor-reduction effect paves the way for curative conversion (ABC conversion) therapy and, therefore, treatment strategies for intermediate-stage HCC may undergo a significant shift in the future. As these treatment strategies are effective in maintaining liver function, even in elderly patients, the transition frequency to second-line treatments could also be improved. These strategies may be effective against nonalcoholic steatohepatitis-related hepatocellular carcinoma and WNT/β-catenin mutations to a certain degree.

## 1. Introduction

**Figure 1:**
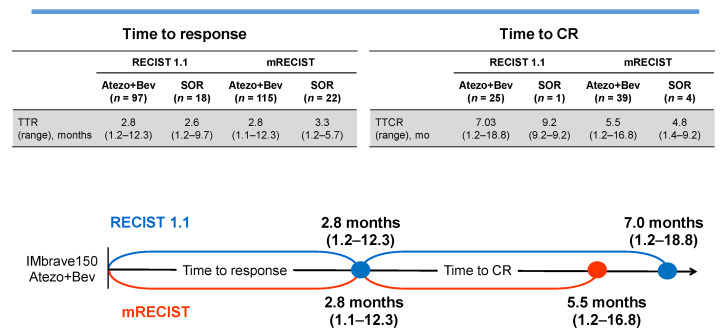
Time to response and time to complete response as per the RECIST v1.1.; modified from [4].

Atezolizumab is an antibody that targets the programmed death ligand 1 (PD-L1), while bevacizumab is an antibody that targets vascular endothelial growth factor A (VEGF-A). The results of a phase 3 trial of atezolizumab plus bevacizumab combination therapy were presented at the November 2019 ESMO Asia Congress [1]. The overall survival (OS) and progression-free survival (PFS) hazard ratios for this combination therapy were 0.58 and 0.59, respectively [1], indicating an outstanding hazard ratio and remarkably superior activity over the multi-kinase inhibitor sorafenib, which had been the standard first-line treatment for advanced hepatocellular carcinoma (HCC) since 2007. Considering the OS and PFS of this therapy, the results of this phase 3 IMbrave150 trial provided a major paradigm-shift in HCC treatment, and atezolizumab plus bevacizumab combination therapy has taken over the standard first-line systemic treatment for advanced HCC for the first time in the past 13 years. Moreover, the median OS of atezolizumab plus bevacizumab combination therapy updated at the 2021 EASL Liver Cancer Summit was 19.2 months, which surpassed the sorafenib median OS of 13.4 months [2]. The response rates were extremely high—30% as per the Response Evaluation Criteria in Solid Tumors (RECIST) v1.1, with 8% complete response (CR). The Kaplan–Meier curve revealed that the OS extension effect of atezolizumab plus bevacizumab combination therapy surpassed sorafenib immediately after the initiation of the treatment. In light of these findings, atezolizumab plus bevacizumab combination therapy was approved in over 70 countries in 2020 as a first-line treatment for advanced HCC [3]. Moreover, studies have also reported the time to response and time to CR based on the results from the phase 3 IMbrave150 trial [4], with a median time to response of 2.8 months (range: 1.2–12.3 months). In addition, the median time to CR was 7 months (range: 1.2–18.8 months) (Figure 1). Some cases reported that partial response (PR) was achieved after 1 year of treatment and CR was reported for the first time after 1.5 years of treatment [4]. Thus, the key to achieving PR/CR is to continue treatment while managing adverse events in patients who present even a slight response or maintain an SD status.

In this review, the paradigm changes in the treatment strategy for HCC due to these practice-changing results of atezolizumab plus bevacizumab combination therapy are discussed in detail, especially the issues concerning several previously unmet medical needs that have been solved by this combination therapy, in addition to the issues that have been raised recently, such as the response of WNT/β-catenin mutation or NASH-HCC to this therapy.

## 2. Phase 1b Arm A Study

In the phase 1b arm A trial, which was a single-arm study assessing the efficacy and safety of atezolizumab plus bevacizumab combination therapy prior to Phase III, the swimmer plot revealed that 17 out of 37 responders achieved CR or PR after two cycles of treatment, with 13 responders achieving the same effect after four cycles [5]. These results indicated that the treatment was highly effective, and a response was quickly achieved in over 80% of the cases in just four cycles (i.e., 12 weeks). Furthermore, these results clearly indicated that there were late responders, illustrated by cases that responded six months after the initiation of the treatments. Thus, these results show that atezolizumab plus bevacizumab combination therapy should be continued in patients whose response maintains a stable disease (SD) or PR status in order to achieve CR.

## 3. Phase 1b Arm F Study

The phase 1b arm F trial was a comparative study to determine whether adding bevacizumab to atezolizumab monotherapy would produce an additive/synergistic effect compared to administering atezolizumab alone [5]. In this study, a hazard ratio of 0.55 was reported, which clearly demonstrated that atezolizumab plus bevacizumab promoted a better PFS than atezolizumab alone. Moreover, the inhibitory effect of bevacizumab on angiogenesis and the change from a suppressive to a responsive immune microenvironment were presumably the main factors that contributed to a better PFS [6]. In fact, at the 2021AACR Annual Meeting, Zhu et al. [7] reported that the effect of bevacizumab plus atezolizumab led to a significantly longer PFS in the Treg high-signature group with a 0.35 hazard ratio (95% CI: 0.15–0.82, *p* = 0.011); however, no significant difference was observed in PFS between the atezolizumab plus bevacizumab combination and atezolizumab monotherapy in the Treg low-signature group (HR 0.82; 95% CI: 0.39–1.7, *p* = 0.64). Similarly, with a hazard ratio of 0.43 (95% CI: 0.19–0.94, *p* = 0.035), atezolizumab plus bevacizumab combination therapy had a significantly better PFS than the single-agent atezolizumab monotherapy in the myeloid-derived suppressor cells (MDSC) high-signature group, thereby demonstrating a clear additive/synergistic effect of bevacizumab. However, no significant difference was observed between the two therapies in the MDSC low-signature group (HR 0.77; 95% CI: 0.36–1.6, *p* = 0.49) [7]. Thus, the findings of the biomarker analysis confirmed that bevacizumab inhibited angiogenesis and transformed the suppressive immune microenvironment into a permissive immune microenvironment [6]. Furthermore, two phase II trials have been conducted to evaluate the direct antitumor effect of bevacizumab monotherapy [8,9]. In the phase II trials conducted by Siegel et al. [8] and Boige et al. [9], the overall response rates (ORR) were 13% and 14%, respectively. Thus, with a 13–14% response rate as per the RECIST v1.0, there is a clear and direct antitumor effect related to the suppression of angiogenesis with bevacizumab monotherapy. In particular, in the phase II trial conducted by Siegel et al., the PFS and OS were 6.9 months and 12.4 months, respectively, which is a similar therapeutic efficacy to that of conventional molecularly targeted agents [10,11,12,13,14]. Therefore, the atezolizumab plus bevacizumab combination therapy produced a synergistic effect through (1) bevacizumab’s direct antitumor effect via inhibition of angiogenesis [8,9]; (2) improvement of the immune microenvironment via bevacizumab’s anti-VEGF effects, such as inhibition of immune suppressive molecules (e.g., Treg, MDSC, or M2-polarized tumor-associated macrophages) [15,16,17,18,19,20]; (3) increased maturation of dendritic cells, increased antigen presentation, and enhanced T-cell infiltration into tumors through vascular normalization as a result of the anti-VEGF effects [21,22]; and (4) the atezolizumab inhibitory effect on immune escape via the PD-1/PD-L1 axis and the reactivation of exhausted T-cells [23].

## 4. Analysis of CR Cases

**Figure 2:**
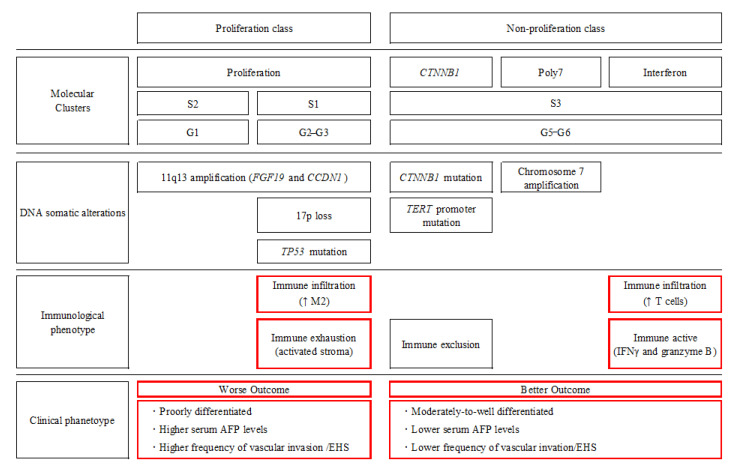
Molecular, immunological, and clinical classification of HCC; modified from [25]. In the clinical phenotype of the worse outcome, an immune-exhaustion class was included for which atezolizumab plus bevacizumab therapy was effective. The immune-active subclass was included in the clinical phenotype of the better outcome. Red box—Important issues. S1, S2 and S3: Molecular Classification by Hoshida. [26]. G1~G6: Molecular classification by Boyaults [27].

In the sub-analysis of the IMbrave150 trial, the background factors of the patient group that achieved CR were examined [1,4,24]. However, this analysis revealed that those who achieved CR did not depend on the presence or absence of typical prognostic factors in the baseline factors, such as the BCLC stage, etiology, AFP value, vascular invasion, and extrahepatic spread; this was because the immune classes were present in both the proliferation and non-proliferation classes within the molecular classification [25] (Figure 2). In other words, the immune classes were present in the groups with poor prognoses (i.e., molecular classes with poorly differentiated hepatocellular carcinoma, high AFP levels, and high vascular invasion), such as those with Hoshida’s S1 subclass [26] and Boyault’s G2–G3 subclasses [27] (e.g., p53 mutation, FGF19 signaling, or abnormal cyclin DNA expression), which are classified as immune exhaustion classes based on immune-based HCC classification [25,28]. These immune exhaustion subclasses do not respond to ICI monotherapy because the immune microenvironment is suppressive, although atezolizumab plus bevacizumab is effective due to the bevacizumab’s effect of transforming the suppressive microenvironment into a responsive microenvironment. Additionally, the nonproliferation classes of Hoshida’s S3 subclass [26] and Boyault’s G5–G6 subclasses [27] belonged to a group with good prognosis, low AFP values, low vascular invasion, and extrahepatic spread [25]. Within this group, part of the interferon expression cluster was categorized as an immune-active class [28] (Figure 2). In this immune-active class, both ICI alone and ICI plus bevacizumab are effective. The immune-exhaustion class has been found to immunotherapy, with the immune microenvironment improving with agents that exhibited an anti-VEGF effect, such as bevacizumab [6,15,16,17,18,19,20]. Thus, regardless of whether the tumor belonged to a proliferation class (i.e., molecular subclass S1) with extremely poor prognosis or a nonproliferation class with good prognosis, the efficacy of atezolizumab plus bevacizumab combination therapy was evident (Figure 2).

## 5. Efficacy in Patients with Vp4

**Figure 3:**
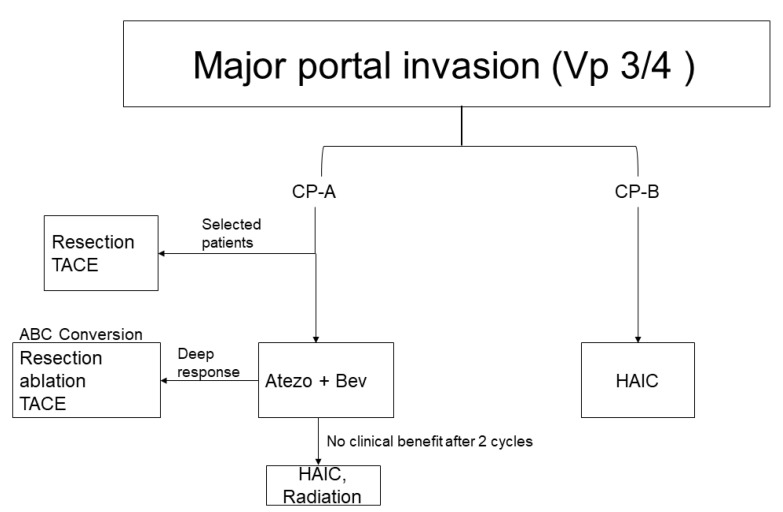
Treatment strategy for advanced HCC with major portal venous invasion. Atezolizumab plus bevacizumab combination therapy could be a first choice of treatment in patients with major vascular invasion and in patients with Child–Pugh class A liver function. Curative conversion can also be expected when deep response is achieved by atezolizumab plus bevacizumab combination therapy (ABC conversion). Vp3, tumor invasion to the first branch of the portal vein; Vp4, tumor invasion into the main trunk of the portal vein; HAIC, hepatic arterial infusion chemotherapy; ABC conversion, atezolizumab plus bevacizumab followed by curative conversion; TACE, transarterial chemoembolization; clinical benefit, AFP level and/or tumor size reduction.

Sub-analysis studies have reported the favorable effects of atezolizumab plus bevacizumab combination therapy in high-risk groups with poor prognoses in IMbrave150, such as those with (1) portal vein invasion at the main portal branch (Vp4), (2) a tumor occupancy rate of over 50%, and (3) bile duct infiltration cases [29]. In particular, the analysis of the Vp4 group was quite interesting because no background differences were observed between groups with or without Vp4 and, except for the Vp4 presence, the OS hazard ratio was similar to the non-Vp4 group (Vp4 group: 0.62, non-Vp4 group: 0.67), where 60% (29/48) of Vp4 patients had extrahepatic spread as well [30]. Moreover, both the groups with and without Vp4 presented equivalent effects on PFS (Vp4 group, PFS HR: 0.62; non-Vp4 group, PFS HR: 0.67). Furthermore, the ORR was 23% and 31% for the Vp4 and non-Vp4 groups, while the CR rate was 4% and 8% for the Vp4 and non-Vp4 groups, respectively. Thus, while the Vp4 group remained slightly inferior to the non-Vp4 group, it exhibited a high response rate that has not been observed with previous molecularly targeted agents [30]. Additionally, while the natural course for Vp4 cases was roughly 3 months [31,32], the use of the atezolizumab plus bevacizumab combination therapy extended that to 7.6 months, which was a clinically meaningful improvement in OS. Traditionally, the treatment strategy in Asian countries for cases with major vascular invasions has been to perform hepatic arterial infusion chemotherapy (HAIC) [33,34,35,36] or radiation therapy [37,38,39] as a first-line treatment, except for selected cases where resection or transcatheter arterial chemoembolization (TACE) is indicated [40,41]. Now, however, the primary treatment strategy would be to administer atezolizumab plus bevacizumab combination therapy, and in case of no response, HAIC could be administered. Therefore, we may expect a major change in the treatment strategies for patients with vascular invasions, including Vp3 and Vp4 (Figure 3).

## 6. Atezolizumab plus Bevacizumab Combination Therapy in Intermediate-Stage HCC

Intermediate-stage HCC, in terms of the Barcelona Clinic Liver Cancer (BCLC) stage, is typically a locally advanced cancer that is not involved in extrahepatic spread or vascular invasion. For intermediate-stage HCC, transarterial chemoembolization (TACE) was previously the standard treatment defined by global guidelines [36,42,43,44]. However, the evidence for TACE was established based on a meta-analysis of six randomized controlled trials compared to nontreatment (best supportive care (BSC)) when no effective systemic agents were available. At present, there are many effective molecularly targeted agents and immunotherapeutic agents, but no established evidence with regard to whether treatment with upfront systemic therapy can provide a better OS benefit than TACE alone. Indeed, some reports have clearly shown that upfront systemic therapy (lenvatinib) followed by super-selective TACE yielded better survival than TACE alone, especially in HCCs beyond the up-to-seven criteria [45]. Thus, the concept of TACE unsuitability has been recently raised at the Asia–Pacific Primary Liver Cancer Expert (APPLE) Consensus [46] and the Clinical Practice Manual for Hepatocellular Carcinoma (JSH consensus) [36]. Moreover, recommendations for systemic therapy over TACE are presently available for those who have (1) tumors with resistance to TACE, such as confluent multinodular-type tumors or poorly differentiated HCC; (2) populations that tend to develop TACE failure, such as those who do not meet the up-to-seven criteria; and (3) those with conditions in which TACE can impair liver function, such as those with modified albumin bilirubin (mALBI) grade 2b [47,48] or among those who do not meet the up-to-seven criteria [49] and have bilobar multifocal nodules. Such recommendations are based on evidence from results seen in (1) sorafenib–TACE sequential therapy [50] and (2) lenvatinib–TACE sequential therapy [45,51]. Sorafenib–TACE sequential therapy clinical trials (the TACTICS trials) revealed that it could prolong PFS but not OS [52]; however, in proof-of-concept trials, a clear OS benefit was shown with LEN–TACE sequential therapy [45]. Furthermore, with numerous validation studies conducted at various institutions [53,54,55,56], the LEN–TACE sequential therapy has become a de facto standard treatment strategy in Japan and Asia for patients with intermediate-stage HCC for which TACE is unsuitable.

**Figure 4:**
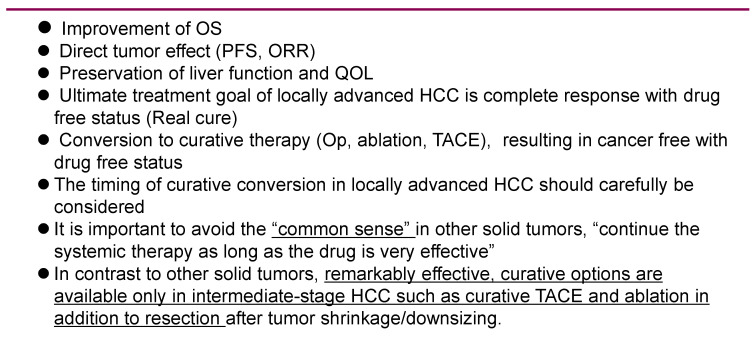
Treatment concept for intermediate-stage hepatocellular carcinoma. HCC— Hepatocellular Carcinoma.

**Figure 5:**
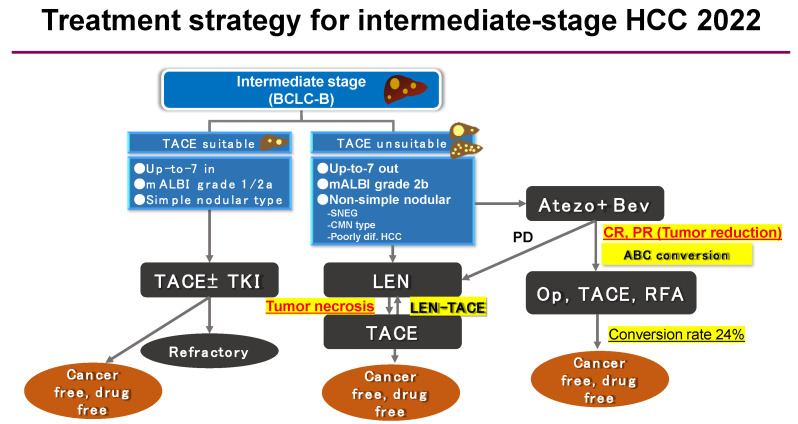
Treatment strategy of intermediate-stage hepatocellular carcinoma. Response of LEN–TACE sequential therapy strongly depends on tumor hypoxic/necrotic change, including with lenvatinib. On the other hand, curative conversion in ABC conversion strongly depends on the tumor shrinkage/reduction effect from atezolizumab plus bevacizumab combination therapy. mALBI grade, modified albumin-bilirubin grade; TACE, transarterial chemoembolization; TKI, tyrosine kinase inhibitor; SNEG, simple nodular type with extra growth; CMN type, confluent multinodular type; ABC conversion, atezolizumab plus bevacizumab followed by curative conversion; LEN, lenvatinib.

Recently, the efficacy of atezolizumab plus bevacizumab combination therapy was shown in patients with BCLC stage B [2]. As a result, the OS, PFS, and ORR for those diagnosed with intermediate-stage HCC who received the atezolizumab plus bevacizumab combination therapy were 25.8 months, 12.6 months, and 44%, respectively. When significant tumor reduction was achieved, indicated by the maintenance of liver function and patient QOL, conversion therapy to curative treatments such as resection, RFA, or curative TACE (atezolizumab plus bevacizumab followed by curative conversion—ABC conversion) was then possible [57,58] (Figure 4 and Figure 5). Through these curative conversions, cancer-free and drug-free statuses were achieved and, according to one report, out of 32 cases that received first-line atezolizumab plus bevacizumab combination therapy, 17 cases were intermediate-stage HCC [57]. The relatively high ratio at this institute of administration of the combination therapy to patients with intermediate-stage HCC was the result of opting for systemic therapy first for the so-called TACE-unsuitable cases, such as those who were beyond the up-to-seven criteria or presented a confluent multinodular type or poorly differentiated type of HCC. Of the 17 cases of intermediate-stage HCC, 6 underwent ABC conversion and were found to be cancer-free and promoted to a drug-free status [58] (Figure 5).

In the field of oncology, the traditional treatment concept for systemic therapy for other types of cancer and even advanced HCC is to continue the administration of the same drugs as long as they are effective (compared to SD). However, unlike other carcinomas, in intermediate-stage HCC it is possible to achieve cancer-free and drug-free status by treating the patient with a curative option, such as resection, ablation, or curative TACE, if a significant tumor reduction is obtained. Therefore, the timing of the initiation of curative conversion should be always carefully considered. As 44% of cases respond to this combination therapy, this indicates that one out of two patients could potentially receive curative conversion (ABC conversion). Since the ultimate treatment goal with a locally advanced HCC is to reach cancer-free or drug-free status, we must exclude the preconceived treatment concept that good effects with the present systemic therapy should be continued with the same agent (Figure 4). Instead, we should switch to the curative therapy when the best response is obtained. For these reasons, it is necessary to develop a approach to systemic therapy for intermediate-stage HCC that is different from that of advanced HCC and other solid tumors (Figure 5).

## 7. Patient-Reported Outcome (PRO)

In the phase 3 IMbrave150 trial, an index called the patient-reported outcome (PRO) was also presented. The PRO is a self-reported, health-related quality of life (HR-QOL) [59] assessment by patients that was undertaken without the involvement of healthcare professionals, as stated in the FDA guidance [60,61]. The PRO index is used as an analysis tool to compare the period until the onset of symptoms. Moreover, the FDA guidance clearly states that the PRO is an important requirement in the drug approval process. In this case, the period until an observed decline in QOL was longer with the atezolizumab plus bevacizumab combination therapy (11.2 months) than sorafenib (3.6 months) (hazard ratio = 0.63 (95% CI: 0.46–0.85)) [59]. Furthermore, the effect of a prolonged QOL correlated with PFS and OS, which indicates that the longer the QOL maintenance, the longer the PFS and OS (PFS HR: 0.93 (95% CI: 0.87–0.99), OS HR: 0.70 (95% CIL 0.65–0.77)) [59].

## 8. Maintenance of Liver Function and Age Analysis

It is widely accepted that monoclonal antibodies can preserve liver function better than tyrosine kinase inhibitors. For example, ramucirumab is a monoclonal antibody that binds to VEGFR2 that does not reduced liver function as determined by the ALBI score when compared with placebo [62]. Moreover, a CheckMate-459 study revealed that nivolumab, an anti-PD-1 antibody, also did not reduce the ALBI score when compared with sorafenib [63]. Similarly, the ALBI score was not impaired in patients that received the atezolizumab plus bevacizumab combination therapy [64]. Therefore, atezolizumab plus bevacizumab combination therapy can efficiently maintain patient liver function and using atezolizumab plus bevacizumab combination therapy as a first-line treatment thus allows for the switch to a second-line treatment while maintaining a Child–Pugh class A liver function, which is important in improving the outcome of sequential systemic therapy [65,66].

Additionally, findings from an age analysis that compared the efficacy and safety between patients ≥65 years old and those <65 years old showed that atezolizumab plus bevacizumab combination therapy was just as effective in elderly patients as the antibody-drug ramucirumab [67]. It was also confirmed that in cases of atezolizumab plus bevacizumab therapy the OS, PFS, and ORR results in elderly patients were comparable to those reported in younger patients [68].

## 9. The Efficacy of Atezolizumab plus Bevacizumab Combination Therapy in HCC with the WNT/β-Catenin Mutation

Previous studies have reported that the efficacy of immune checkpoint inhibitor (ICI) monotherapy was ineffective in cases where patients exhibited the WNT/β-catenin activating mutation [69,70]. The presence of the WNT/β-catenin mutation can be evaluated in the hepatobiliary phase of gadolinium-ethoxybenzyl-diethylenetriamine pentaacetic acid-enhanced MRI (Gd-DTPA-EOB MRI) [71,72]. Recently, this hepatobiliary phase of EOB-MRI was reported to act as an imaging biomarker for predicting poor treatment response to PD-1/PD-L1 antibody therapy [73]. Importantly, this was only a therapeutic efficacy prediction marker for ICI monotherapy; however, whether the combination of atezolizumab plus bevacizumab was effective in patients with the WNT/β-catenin mutation was not evaluated and remains to be elucidated with the accumulation of more cases in real-world clinical practice. Indeed, atezolizumab plus bevacizumab combination therapy was reportedly effective in one patient with the WNT/β-catenin mutation [74], presumably due to the release of a tumor antigen that resulted from tumor necrosis caused by bevacizumab’s direct antitumor effect (bevacizumab ORR = 13–14% in 2 phase II studies of bevacizumab monotherapy [8,9]). This in turn activated CD8-positive cells via the presentation of cancer antigens by MHC class 1 molecules in matured dendritic cells. In addition, bevacizumab normalized tumor blood vessels, which led to invasion of activated CD8-positive cells into the HCC. Thus, these results indicate cases in which a level of effectiveness could be expected even in the presence of the WNT/β-catenin mutation. Nevertheless, evaluation of more clinical cases with the WNT/β-catenin mutation is required to draw conclusions on whether the combination therapy is effective for patients with this mutation.

## 10. The Efficacy of Atezolizumab plus Bevacizumab Combination Therapy against NASH-HCC

Recent studies published in *Nature* reported that in nonalcoholic steatohepatitis-related hepatocellular carcinoma (NASH-HCC) mouse models, resident-like CXCR6-positive and CD8-positive cells were activated rather than T-cells through MHC class 1 molecule antigen presentation, which promoted a poor immunotherapy response [75,76]. Moreover, the aforementioned clinical studies reported meta-analyses of the results from three phase 3 immunotherapy trials (CheckMate-459 [77], Keynote 240 [78], and IMbrave150 [1]) and findings from two validation cohort studies that suggested that monotherapies with immunotherapy agents elicit poor OS. However, these validation results were obtained from patients who received only PD-1/PD-L1 antibody monotherapy. In particular, the OS HR in HCC with nonviral etiology in an IMbrave150 trial was 1.05, which is seemingly less effective than sorafenib. However, the OS of the atezolizumab plus bevacizumab combination therapy group was 17.0 months, which is comparable to the OS of HBV HCC (19.0 months). Thus, the atezolizumab plus bevacizumab combination therapy may have therapeutic effects on HCC with nonviral etiology.

Conversely, the OSs of the sorafenib group for nonviral HCC, HBV HCC, and HCV HCC were 18.1, 12.4, and 12.6 months, respectively. Therefore, in HCC with a nonviral etiology, the OS HR of 1.05 seemed to be a result of the excellent efficacy of sorafenib, although the reason behind the high efficacy of sorafenib remains unclear. It is widely accepted that, since even mild fibrosis can lead to cancer incidence with NASH/NAFLD-HCC (Table 1) [79,80,81,82,83], liver function may be maintained and lead to favorable effects with sorafenib treatment. Good liver function also allows for post-progression treatment and contributes to the OS extension effect.

The previous meta-analyses were not limited to NASH/NAFLD, while in this analysis, all HCCs with nonviral etiology were included as one group. Since early phase studies of ICI reported that HCC of nonviral etiology is most responsive to nivolumab [84], pembrolizumab [85], and nivolumab plus ipilimumab [86] therapies, based on the waterfall plots or ORRs, HCC of nonviral etiology as a whole should be considered separately from NASH/NAFLD-derived HCC. The published *Nature* article stated that “therapeutic PD1- or PD-L1-related immunotherapy failed to cause tumor regression in NASH–HCC” [76]; this statement did not apply to HCC with nonviral etiology as a whole, but to NASH-HCC only. Furthermore, the direct antitumor effect of atezolizumab plus bevacizumab against HCC with nonviral etiology indicated a PFS of 7.1 months and an ORR of 27% [2].

The findings from the phase 1b arm F study for atezolizumab plus bevacizumab combination therapy provide better insight into its improved efficiency in treating HCC with nonviral etiology when compared with ICI monotherapy. In the nonviral cases, the PFS hazard ratio was 0.49, and better results were obtained with atezolizumab plus bevacizumab than with atezolizumab monotherapy. Therefore, in addition to the positive impact of bevacizumab on the immune microenvironment and its direct antitumor effect (ORR: 13%–14%), bevacizumab may further enhance this therapeutic effect by inhibiting tumor growth, triggering tumor antigen production, promoting dendritic cell maturation, presenting cancer antigens to T cells, and stimulating tumor infiltration by activated CD8-positive cells during the cancer immunity cycle [23]. Therefore, it is necessary to continue accumulating cases that report the effects of atezolizumab plus bevacizumab on NASH-HCC in real-world clinical practices. For example, at our institute, two of the 15 NASH-HCC cases (nine biopsy-proven and six clinically diagnosed NASH-related HCC cases) responded successfully to the combination therapy (Table 2). From these factors, the conclusions with regard to the treatment strategy using atezolizumab plus bevacizumab combination therapy in relation to NASH-HCC can be summarized as follows:

1. Pure NASH-HCC may not respond to ICI monotherapy because it does not provide antigens presenting activation of CD8-positive cells by MHC class 1 molecules;
2. The therapeutic efficacy of combined atezolizumab plus bevacizumab treatment in NASH-HCC cases requires further investigation of real-world clinical data;
3. Definitive diagnosis is difficult for NASH-HCC without histological diagnosis under routine clinical practice. Thus, diagnosis of NASH-HCC based solely on clinical findings may not indicate a pure NASH-HCC condition;
4. Making a clinical diagnosis without including histological confirmation could be a disadvantage for patients because it would exclude atezolizumab plus bevacizumab combination therapy treatment. If pathology results do not confirm NASH-HCC, then treatment should commence with first-line atezolizumab plus bevacizumab therapy; however, if this treatment is ineffective, then patients should be immediately transitioned to the next line of treatment. Thus, when initiating treatment, NASH-HCC should be identified with an understanding that atezolizumab plus bevacizumab may not be effective in some cases.

In contrast to ICI, previous studies have reported that lenvatinib is equally effective in NASH-HCC with other etiologies [87] and more effective against NASH/NAFLD-associated HCC than against HCC with alcohol or viral etiologies [88]. Therefore, when a patient is clinically suspected to present NASH-HCC and does not respond to atezolizumab plus bevacizumab therapy, the treatment should be immediately switched to lenvatinib or another therapy associated with improved disease status. Lenvatinib after immunotherapy was more effective than when it was used as first-line therapy [89]; therefore, it can be expected that lenvatinib could have a better efficacy when administered after PD with atezolizumab plus bevacizumab therapy than as an initial treatment strategy.

## 11. Conclusions

Atezolizumab plus bevacizumab combination therapy is hypothesized to improve the prognosis of patients with advanced HCC [90,91,92]. In addition, the safety of atezolizumab plus bevacizumab in real-world practice is consistent with that in the phase 3 IMbrave150 trial [90]. This therapy can increase the number of advanced HCC conversions to resections. Moreover, in patients with HCC at the TACE-unsuitable intermediate stage, atezolizumab plus bevacizumab combination therapy is a potential treatment choice. If significant tumor reduction is achieved in such patients, curative conversion (ABC conversion) could be applied. Indeed, the purpose of treating locally advanced intermediate-stage HCC is to achieve cancer-free and drug-free status, which benefits patients. Therefore, it is crucial not to miss the best timing for curative conversion (usually at the best response). While several clinical trials are underway, success in adjuvant and intermediate-stage clinical trials will presumably change the treatment strategy and landscape in HCC drastically. Currently, however, a treatment strategy based on atezolizumab plus bevacizumab combination therapy still plays an extremely important therapeutic role for TACE-unsuitable intermediate-stage and advanced-stage HCC.

## Figures and Tables

**Table 1:** Presence of liver cirrhosis at the procarcinogens.

	NAFLD	HCV	HBV	Alcohol Abuse	Idiopathic
LC (%)	65.4	91.1	92.3	88.9	66.2
non LC (%)	34.6	8.9	7.7	11.1	33.8

LC, liver cirrhosis. Modified form [78]. NAFLD, Non-alcoholic fatty liver disease; HCV, hepatitis C virus; HBV, Hepatitis B virus.

**Table 2:** Atezolizumab + Bevacizumab: ORR by Etiology (RECIST v1.1). *N* = 77.

Response Category	HCV (*N* = 24)	HBV (*N* = 16)	NBNC_alcohol (*N* = 18)	NBNC_NASH (*N* = 17)	*p* Value
**ORR**	**33.3% (8/24)**	**25.0% (4/16)**	**27.8% (5/18)**	**11.8% (2/17)**	**N.S.**
**DCR**	**75.0% (18/24)**	**68.8% (11/16)**	**77.8% (14/18)**	**35.3% (6/17)**	**0.028**
CR	0	0	0	0	
PR	8	4	5	2	
SD	10	7	9	4	
PD	5	5	3	11	
NE	1	0	1	0	

ORR, objective response rate; DCR, Disease control rate; CR, complete response; PR, partial response; SD, stable disease; PD, progressive disease; NE, not evaluable. Cases from Kindai University Hospital.
